# Revisiting factors associated with blood culture positivity: Critical factors after the introduction of automated continuous monitoring blood culture systems

**DOI:** 10.1097/MD.0000000000029693

**Published:** 2022-07-29

**Authors:** Pei-Chin Lin, Chia-Ling Chang, Yi-Hua Chung, Chih-Chun Chang, Fang-Yeh Chu

**Affiliations:** a Department of Clinical Pathology, Far Eastern Memorial Hospital, New Taipei City, Taiwan; b Department of Nursing, Cardinal Tien Junior College of Healthcare and Management, Yilan, Taiwan; c Graduate School of Biotechnology and Bioengineering, Yuan Ze University, Taoyuan City, Taiwan; d Department of Medical Laboratory Science and Biotechnology, Yuanpei University of Medical Technology, Hsinchu City, Taiwan; e School of Medical Laboratory Science and Biotechnology, Taipei Medical University, Taipei City, Taiwan.

**Keywords:** automated continuous monitoring blood culture system, blood culture, blood culture set, blood culture volume, diagnosis of sepsis, positivity rate

## Abstract

Blood culture is the main tool used to identify causative pathogens. Adequate volume and number of culture sets are considered key to blood culture positivity rate. It is not known whether these factors remain critical to the positivity rate after the introduction of automated continuous blood culture system monitoring.

We measured blood volume per bottle and described the distribution of blood volume and number of culture sets. Multivariate logistic regression was performed to determine the independent association of blood volume, number of culture sets, diagnosis of sepsis in a patient, and other covariates with blood culture results.

Only 6.9% of the blood culture bottle volumes complied with the guidance (8–10 mL), with the highest culture positivity rate (18%). Of the culture events, only one set of blood was cultured in 60.9% of events. In the multivariate analysis, blood culture volume per event (odds ratio [OR], 1.09 [95% confidence interval [CI], 1.06–1.11]), patients with a diagnosis of sepsis (OR, 2.86 [95% CI, 2.06–3.98]), and samples from the emergency department (OR, 2.29 [95% CI, 1.72–3.04]), but not the number of culture sets (OR, 0.74 [95% CI, 0.50–1.12]), were observed to be statistically significant with respect to blood culture positivity rate.

Our results revealed that the total blood culture volume and the diagnosis of sepsis were critical factors affecting blood culture positivity rate. However, the proportion of blood culture bottles with the optimal blood volume was very low, and optimizing blood volume would be key to increasing blood culture positivity rate.

## 1. Introduction

Bloodstream infection (BSI) is a global problem that can result in serious morbidity and mortality. Early detection of bacteremia followed by pathogenic microorganism identification and determination of antimicrobial susceptibility are important for guiding antibiotic therapy. The mortality rate of patients receiving appropriate therapy is considerably lower than that of patients treated with ineffective antibiotics.^[1]^

Blood culture is the main tool used to identify causative pathogens.^[2]^ Various factors affect the positivity rate of blood culture, including the timing of sample collection, skin antiseptic preparation, number of blood culture sets, and blood volume inoculated in individual culture bottles.^[3]^ Cultures obtained before antimicrobial therapy are recommended because antibiotic administration may interfere with bacterial growth.^[4]^ Collection of at least 2 blood culture sets within a 24-hour period is recommended for the detection of BSI in adult patients.^[2,3]^ However, only one blood culture set was collected from some patients with suspected BSI in Taiwan.^[5]^ Adequate blood volume sampling is also crucial for positivity rate, with a 2% to 4% increase in positivity rate from each additional milliliter of blood.^[2]^ In adults, a blood culture volume of 8 to 10 mL per bottle is recommended, and the limit of acceptable volume is 3 mL per bottle.^[6,7]^ According to the microbiology checklist of the College of American Pathologists, one set of blood culture bottles for adults should have at least 20 mL of blood (including 10 mL aerobic and 10 mL anaerobic bottles).^[8]^ However, the blood volume in each culture bottle is generally low in Taiwan.^[9]^ A previous study also revealed that 40% to 85% of the collected blood culture volume was inadequate.^[2]^

With the development of blood culture technology, bacterial growth can be detected by automated continuous monitoring of blood culture systems, which is a more sensitive approach than the traditional method and shortens blood culture turn-around time.^[10]^ The BACTEC™ FX system (BD, Sparks, MD) can estimate blood culture volumes in blood culture bottles based on red blood cell metabolism.^[11]^ Other new technologies such as molecular biology techniques and mass spectrometry have also been developed to improve the identification of pathogens in blood culture.^[12–14]^ It remains unknown whether blood culture volume and number of culture sets, which were considered important in the past, are still critical for positivity rate after the introduction of these modern methods for blood culture.

The aim of this study was to analyze the effect of blood culture volume and the number of culture sets on the positivity rate of blood cultures in the era of automated continuous monitoring of blood culture systems. The blood culture bottle type, sampling department, and diagnosis of sepsis were also investigated. Furthermore, we describe the distribution of blood volume and number of culture sets and compare the difference between the estimated blood culture volume using the BACTEC™ FX system and the actual measured blood culture volume.

## 2. Materials and Methods

### 2.1. Research design

In this retrospective study, we analyzed all blood cultures collected at the emergency department (ED), intensive care unit (ICU), general ward (GW), and outpatient department in Far East Memorial Hospital (FEMH) from March 1, 2020 to March 31, 2020. Pediatric blood culture bottles, fungus blood culture bottles, and blood cultures with incomplete data were excluded.

Data on blood culture bottle type, sampling time, and sampling department for all individual blood culture bottles were collected from the laboratory information system of the FEMH. Patients diagnosed with sepsis were identified using *International Classification of Diseases* (*ICD*)-9 or *ICD-10* diagnostic codes and further confirmed by a chart review according to the sepsis-3 diagnostic criteria.^[15]^ Four types of blood culture bottles were used in this study, namely, BACTEC™ Lytic/10 Anaerobic/F Culture Vials, Plus Aerobic/F Culture Vials, Plus Anaerobic/F Culture Vials, and Standard/10 Aerobic/F Culture Vials (BD, Baltimore). The blood culture set included an aerobic bottle and an anaerobic bottle. The indication for blood culture was determined by the attending physician based on the clinical presentation of the patient. After collection, all blood cultures were transferred to the laboratory within 2 hours and were incubated in an automated continuous monitoring blood culture system (BACTEC™ FX system; BD, Sparks, MD) until a positive signal or for a period of 5 days. Gram staining of blood culture bottles with a positive signal was performed to confirm them as true positives. The standard method for identification and susceptibility testing was then performed, including matrix-assisted laser desorption ionization-time of flight mass spectrometry (Bruker Daltonik, Bremen, Germany). If there was no positive signal after 5-day incubation, the blood culture bottle was considered negative. Approval was obtained from the ethics committee of FEMH (approval number 110089-E). The requirement for written informed consent was waived owing to the retrospective nature of the study and the use of deidentified data. All procedures used in this study adhere to the tenets of the Declaration of Helsinki.

### 2.2. Determination of blood culture contamination rate

The overall contamination rate of blood cultures was calculated based on previously published data.^[16,17]^ A potentially contaminated blood culture was defined as yielding normal skin flora bacteria, including coagulase-negative *Staphylococci*, *Bacillus* spp., *Corynebacterium* spp., and *Micrococcus* spp. For these microorganisms, at least 2 positive blood culture sets yielding the same results in 24 hours were considered clinically significant.

Definition of blood culture volume (actual blood volume [AV] and virtual blood volume [VV]).

The AV in each culture bottle was calculated using the following formula:


$$\begin{array}{l} \text{Blood volume (mL) }=\frac{[\text{ weight of the blood bottle after sampling (g)}-\text{ empty blood bottle weight(g) }]}{\text{blood density (1}\text{.055g / mL)}} \end{array}$$

Each blood culture bottle was weighed at 0.1 g with an electronic scale. Furthermore, we defined the blood culture volumes estimated using the BACTEC™ FX system as VV in this study. The BACTEC™FX system was able to estimate the mean blood culture volume of Plus Aerobic/F media bottles with negative culture results. The mean VV from each sampling department was collected using the BD EpiCenter™ microbiology data management system.^[11]^

### 2.3. Statistical methods

Descriptive statistics were used to summarize the data. Categorical data are reported as percentage and continuous data are reported as median with interquartile range or mean with standard deviation. Differences in ≥2 groups were analyzed using the nonparametric Wilcoxon rank-sum test or Kruskal–Wallis test and post hoc analysis with Bonferroni correction, respectively. To compare AV and VV, Spearman correlation coefficients and *t* tests were performed. Blood cultures collected from the same patient on the same day were defined as the same blood culture event. The number of blood culture sets was calculated based on each blood culture event. Qualitative data were analyzed using the χ^2^ or Fisher exact test. Multivariate logistic regression was performed to determine the independent association of blood culture volume, number of blood culture sets, and other covariates with blood culture results. Statistical significance was set at *P* < .05. Data were analyzed using IBM SPSS Statistics for Windows (version 19, IBM Corp., Armonk, NY).

## 3. Results

### 3.1. Characteristics of blood cultures in the study

From March 1, 2020 to March 31, 2020, a total of 3256 blood culture sets were issued in the FEMH. After excluding pediatric blood culture bottles, fungus blood culture bottles, and incomplete data registration, there were 2866 sets of blood cultures (5732 blood culture bottles). Considering blood cultures from the same patient on the same day, there were 2023 blood culture events. Among them, 257 events (12.7%) were from patients with a diagnosis of sepsis and 1766 events (87.3%) were from patients without sepsis (Table 1). A total of 837 blood culture events (41.4%) were ordered when patients were in the ED and 780 culture events (38.6%) in the GW. There were 792 blood culture events (39.1%) with >1 set of blood cultures and 1232 (60.9%) events with only a single set of blood cultures. A total of 13.7% of all blood culture events were positive, with a higher positivity rate found in the group of patients diagnosed with sepsis (26.5% vs 11.9%).

**Table 1:** Characteristics of culture events in patients diagnosed with or without sepsis.

	Nonsepsis (N = 1766)	Sepsis (N = 257)	*P* value
**Age**	64 (IQR, 50–76)	69 (IQR, 58–80)	<.001
**Sex (female, %**)	772 (43.7%)	111 (43.2%)	.87
**Septic shock**	NA	158 (61.5%)	NA
**Collection department**			<.001
ED	763 (43.2%)	74 (28.8%)	
ICU	290 (16.4%)	63 (24.5%)	
OPD	51 (2.9%)	2 (0.8%)	
GW	662 (37.5%)	118 (45.9%)	
**Number of blood culture sets**			.002
1	1092 (61.8%)	139 (54.1%)	
2	646 (36.6%)	107 (41.6%)	
3	21 (1.2%)	6 (2.3%)	
4	7 (0.4%)	5 (1.9%)	
**Total culture volume per event (mL**)	12.8 (IQR, 9.5–19.9)	13.3 (IQR, 10.4–20.4)	.16
**Positive blood culture events (positivity rate**)	210 (11.9%)	68 (26.5%)	<.001

### 3.2. Distribution of microorganisms isolated from the positive blood culture

The distribution of microorganisms isolated from the positive blood culture sets is shown in Table 2. *Escherichia coli* was the most commonly occurring Gram-negative bacterium, followed by *Klebsiella pneumoniae* and *Pseudomonas aeruginosa*. Coagulase-negative *Staphylococcus and Staphylococcus aureus* occurred most commonly in Gram-positive bacterial isolates. The overall blood culture contamination rate was 0.87% during the study.

**Table 2:** Distribution of microorganisms isolated from positive blood culture sets.

Microorganism	Number	%
**Gram-positive bacteria**	244	54.3%
Coagulase-negative staphylococci	77	17.1%
*Staphylococcus aureus*	63	14.0%
*Streptococcus mitis group*	24	5.3%
*Bacillus species*	10	2.2%
*Enterococcus faecium*	9	2.0%
*β-Streptococcus group B*	9	2.0%
*β-Streptococcus group G*	8	1.8%
*Enterococcus faecalis*	7	1.6%
Other Gram-positive bacteria*	37	8.2%
**Gram-negative bacteria**	183	40.8%
*Escherichia coli*	69	15.4%
*Klebsiella pneumoniae*	46	10.2%
*Pseudomonas aeruginosa*	11	2.4%
*Proteus mirabilis*	7	1.6%
*Acinetobacter baumanii complex*	6	1.3%
*Enterobacter cloacae complex*	5	1.1%
Other Gram-negative bacteria†	39	8.7%
**Yeasts**	22	4.9%
*Candida albicans*	7	1.6%
*Candida parapsilosis*	7	1.6%
*Candida glabrata*	5	1.1%
Other yeasts‡	3	0.7%
**Total**	449	100.0%

### 3.3. Detection of growth in relation to blood culture set number

**Figure 1.**
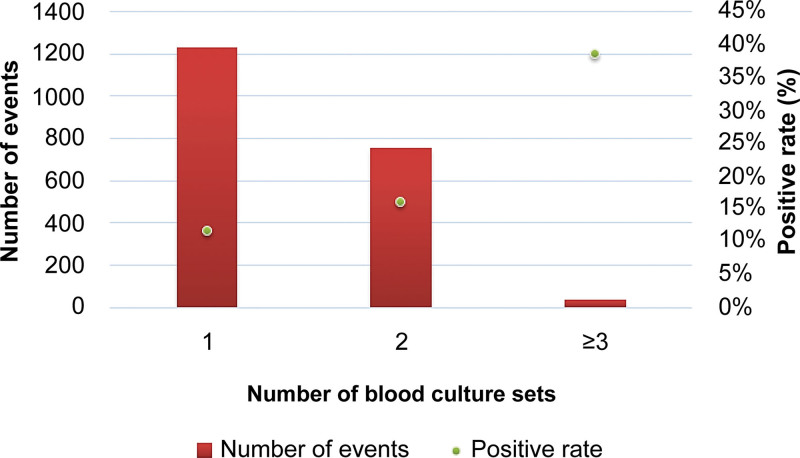
Blood culture positivity rate and sampling set distribution (per event).

The association between the number of blood culture sets and positivity rate in the 2023 blood culture events is shown in Figure 1. There were 1231 blood culture events (60.9%) in which one blood culture set was collected with a mean total blood culture volume of 10.6 ± 3.6 mL (≈5.3 mL of blood per bottle) and a positivity rate of 11.5%. There were 753 events (37.2%) in which 2 blood culture sets were collected with a mean total blood volume of 21.3 ± 5.3 mL (≈5.3 mL of blood per bottle) and a positivity rate of 16.1%. There were 39 events (1.9%) in which ≥3 sets were collected with a mean total blood volume of 35.4 ± 8.1 mL (≈5.8 mL of blood per bottle) and the highest positivity rate of 38.5%.

### 3.4. Detection of growth in relation to blood volume collected into culture bottles

**Figure 2.**
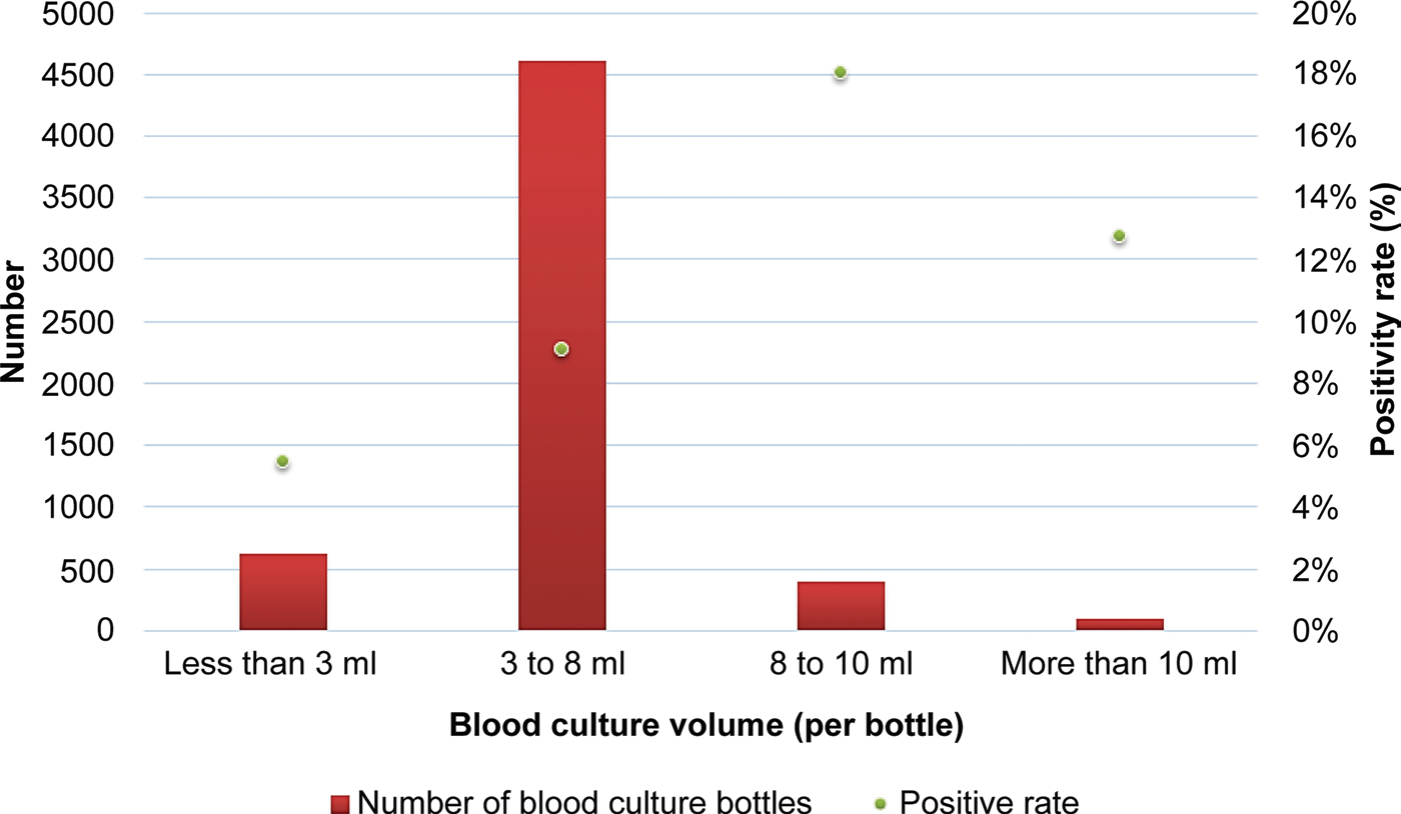
Blood culture positivity rate and volume distribution (per bottle).

**Figure 3.**
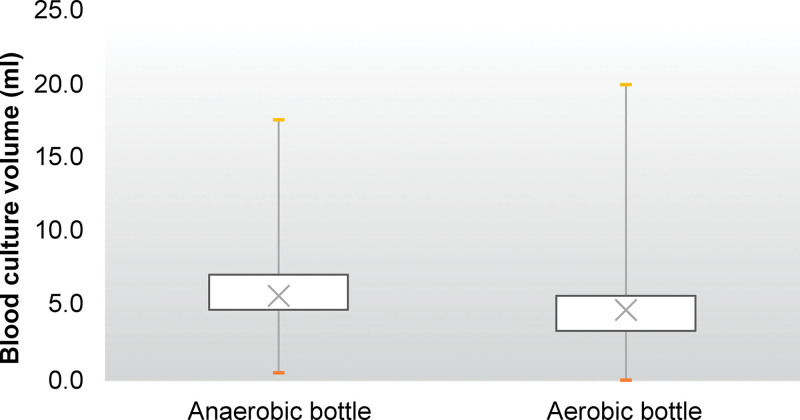
Box-and-whisker plot of blood culture volume (mL) based on culture bottle—anaerobic bottle vs aerobic bottle.

Of the 5732 blood culture bottles, 538 (9.4%) were positive. Only 6.9% of the blood culture bottles were filled with 8 to 10 mL of blood, where the positivity rate of 18.1% was the highest. In 80.5% of the blood culture bottles, 3 mL to 8 mL of blood was collected, with a positivity rate of 9.1%. In 10.8% of bottles, <3 mL of blood was collected, with the lowest positivity rate of 5.5%; in 1.8% of blood culture bottles, more than 10 mL of blood was collected, with a positivity rate of 12.7% (Fig. 2). The median sampling volume in anaerobic bottles was higher than that in aerobic bottles, which was 5.7 and 4.7 mL, respectively (*P* < .001) (Fig. 3). It was found that 81% of anaerobic bottles had a higher blood culture volume than aerobic bottles.

### 3.5. Multivariate analysis of factors associated with blood culture positivity

The multivariate analysis showed a statistically significant interaction between the total blood culture volume per event and positive culture results (odds ratio [OR], 1.09; *P* < .001). Moreover, patients with a diagnosis of sepsis (OR, 2.86, *P* < .001) and sampling from the ED (OR, 2.29; *P* < .001) were observed to be statistically significant. However, the number of sets with > 1 set was associated with positive culture results in the univariate analysis (OR, 1.59, *P* < .001) but lost statistical significance after the multivariate analysis (OR, 0.74, *P* = .15) (Table 3).

**Table 3:** Multivariate analysis of factors affecting positive blood culture events.

Factor	Odds ratio (95% CI)	*P* value
**Age (yr**)	1.01 (1.00–1.02)	.004
**Gender (male vs female**)	0.96 (0.74–1.25)	.77
**Sepsis**	2.86 (2.06–3.98)	<.001
**Sampling department(ED vs non-ED**)	2.29 (1.72–3.04)	<.001
**Total volume of collection (mL**)	1.09 (1.06–1.11)	<.001
**Number of cultures with >1 set**	0.74 (0.50–1.12)	.15

### 3.6. Comparison of VV using the BD BACTEC™ FX system with AV measured manually

**Figure 4.**
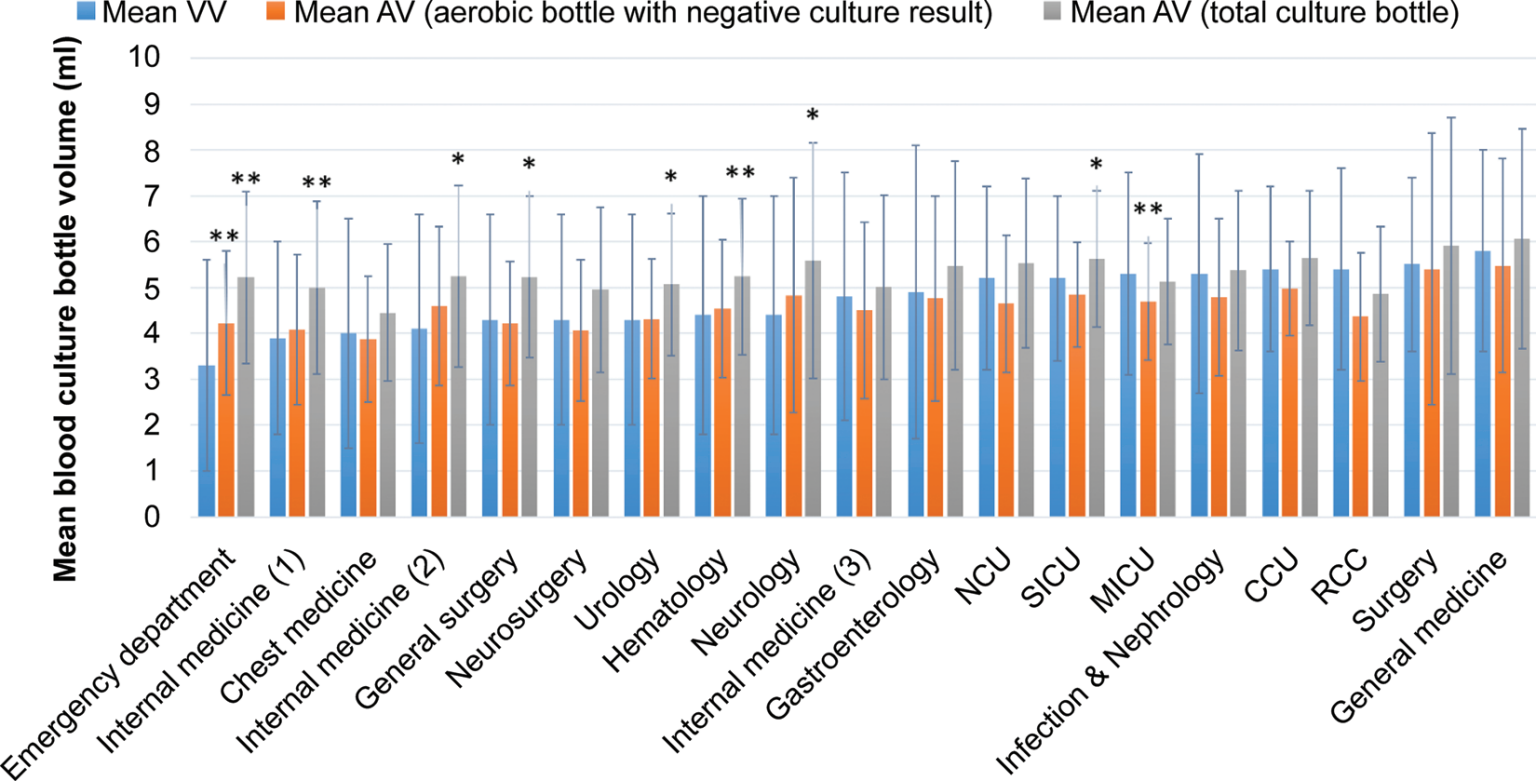
Comparison of mean VV of aerobic/F bottles estimated using the BACTEC™ FX system with AV measured manually in different sampling departments (departments that collected ≥ 25 blood culture sets were included). *P < .05 (compared with VV). **P < .01 (compared with VV). AV = actual blood volume, CCU = cardiac intensive care unit, MICU = medical intensive care unit, NCU = neurological intensive care unit, RCC = respiratory care center, SICU = surgical intensive care unit, VV = virtual blood volume.

The comparison between mean VV estimated using the BACTEC™ FX system and AV in different sampling departments is shown in Figure 4. The mean AV of total blood culture bottles was 5.3 ± 2.0 mL (*P* < .001). The mean AV of aerobic bottles with negative culture results was 4.5 ± 1.8 mL (*P* = .004). The mean VV of aerobic/F bottles from the EpiCenter data was 4.7 ± 2.3 mL. There was a significant difference between AV and VV in the 9 sampling departments. The estimated VV was significantly lower than the AV in the ED, internal medicine, general surgery, urology, hematology, neurology, and surgical ICUs. The VV was higher than the AV in the medical ICU. The correlation between VV and AV was analyzed in the 19 sampling departments that submitted more than 25 blood culture sets. There was a positive correlation between the VV from the BACTEC™ FX system and the AV of aerobic bottles with negative culture results (*R* = 0.761, *P* < .001) and between the VV from the BACTEC™ FX system and the AV of total blood culture bottles (*R* = 0.568, *P* = .011).

## 4. Discussion

In the present study, we studied the factors that may contribute to blood culture positivity rate, including the diagnosis of sepsis, blood culture volume, number of blood culture sets, and sampling department. We found that total blood culture volume per event, diagnosis of sepsis, and sampling in the ED were statistically significant factors for positive culture results as determined using the multiple logistic regression analysis. A previous study also demonstrated that the total blood culture volume was a significant factor for culture positivity rate after multivariate analysis with an OR of 1.02 (95% CI, 1.01–1.03, *P* < .001).^[18]^ Following the recommendation to collect at least 2 blood culture sets and a blood culture volume of 8 to 10 mL per bottle, the optimal total blood culture volume was estimated to be 30 to 40 mL.^[2,3,8]^ However, we found that the total blood culture volume was inadequate for most culture events. This could be attributed to the fact that only one culture set was collected in most culture events, and inadequate blood volumes were sampled per bottle.

In 80.5% of blood culture bottles, <8 mL of blood was collected. Furthermore, in 10.8% of blood culture bottles, even <3 mL of blood was collected. These findings were similar to those of previous studies in Taiwan and South Korea, which also indicated that in most blood culture bottles, blood was collected at less than the recommended volume of 8 to 10 mL.^[9,19]^ The group with blood volumes of <3 mL showed the lowest positivity rate. As the blood volume increased, the positivity rate also tended to increase. However, when the blood volume was >10 mL, there was no increase in the positivity rate. This result was consistent with that of a previous study in 2013.^[9]^ This suggests that too much blood would dilute the designed default concentration in the blood bottle. We also found that the blood volume in anaerobic bottles was significantly higher than that in anaerobic bottles. This phenomenon has also been observed in a previous study, and it may be related to the sampling order in which the bottles were collected first.^[19]^ Besides, insufficient knowledge about optimal blood culture volume and the limitations of blood collection devices may be the reasons for inadequate blood culture volume per bottle. Several medical personnel consider that only 3 to 7 mL of blood is sufficient for an individual blood culture bottle. In addition, a majority of medical personnel used 10-mL syringes for blood culture collection, which may explain the low blood culture volume and the uneven distribution of volume in anaerobic and aerobic bottles. With an optimal blood volume of 20 mL per set, 10 mL of blood did not meet the recommendation. In addition, physicians usually ordered blood cultures along with other blood tests. Thus, the blood in a 10-mL syringe was distributed into multiple tubes, which may cause less blood to be collected in the culture bottles.

Most interestingly, previous studies demonstrated the influence of blood culture sets on the positivity rate.^[20]^ In our study, we also found that more blood culture sets had a higher positivity rate. However, the number of blood culture sets was not associated with positive culture results in the multivariate analysis in our study. This may imply that the increase in the positivity rate seen in higher culture set numbers could largely be attributed to the higher total culture volume. This could be correlated to the fact that most BSI episodes in adults present <1.0 CFU/mL of microorganisms in the blood,^[21]^ and therefore, the adequate blood volume was still the key to the positivity rate. However, the number of culture sets was still important because it was necessary to collect more culture sets to obtain a higher total blood volume, with an optimal blood volume per bottle of 8 to 10 mL. Furthermore, previous studies showed that the positivity rate of one set of blood cultures was ≈80%, the positivity rate of 2 sets was nearly 90%, and for 3 sets it reached almost 99%.^[20]^ However, the blood culture positivity rate in the present study was low. Possible reasons include that although many sets of blood cultures were collected, the blood culture volume in each bottle was still low. The uneven distribution between the aerobic and anaerobic bottles is also a possible reason. Even though the blood volume collected was as high as 20 mL, its uneven distribution in different bottles might cause the positivity rate to change. Furthermore, patients diagnosed with sepsis and blood culture sets collected in the ED were significant factors for positivity rate. This may be because the condition of the ED patients was more serious, and most of them may have blood cultures sampled before antibiotic administration. These findings should be validated with further research.

The overall VV was significantly lower than the AV of all blood culture bottles, and the most obvious difference was found in the ED. This may be because the BACTEC™FX system could only detect Plus Aerobic/F media bottles (an aerobic blood culture bottle with resin, which is applied to patients who already had antibiotic administration^[22]^) with negative culture results. First, if a department has more blood culture bottles without resin, which will not be included in detection using the BACTEC™FX system, then it might cause greater difference between VV and AV. For example, most patients in ED are not administered antibiotics when collecting blood culture, and therefore, blood culture bottles without resin are often used. Moreover, these culture bottles will not be detected using the BACTEC™FX system. This may be the reason for a greater difference between VV and AV in ED than in other departments. Second, blood culture bottles with positive culture results usually have a higher blood volume. However, the BACTEC™FX system cannot detect the volume of positive culture bottle, and this may also cause VV underestimation. Furthermore, BACTEC™FX system only detects aerobic blood culture bottles. Our study found aerobic bottles with lower blood volume than anaerobic bottles, which may also cause VV underestimation. Finally, a previous study demonstrated imprecision in VV when the hematocrit value of patients was below 30%.^[23]^ However, the hematocrit value was not collected in our study, which needed further research. These all may be the potential reasons for the significant difference between AV and VV in different departments. Furthermore, some studies have applied AV data from the BACTEC™FX system as an index to monitor blood culture volume in hospitals.^[2,24]^ Although it may be a substitute, the BACTEC™FX system cannot reflect the real problem in blood culture collection, as it only offers the average blood volume of the sampling department. We could not determine the number of blood culture bottles with a volume lower than the recommended volume. For example, if a sampling department had a few blood culture bottles with over 10 mL of blood, and others with <3 mL, its average blood volume may be within 8 to 10 mL. However, our results revealed that the excess or inadequate blood volume in each bottle resulted in a lower culture positivity rate.

Our study had some limitations. This was a single-center retrospective study, and the sample size was relatively small. Additionally, the potentially contaminated blood culture sets were not excluded, which could lead to certain bias. However, the effect on the results may be limited due to a low contamination rate in our hospital. Blood cultures from pediatric patients were excluded, and therefore, the results of this study could not be applied to the pediatric population. However, in this study, we investigated the effect of several factors on blood culture positivity rate, including blood culture volume, set number, the diagnosis of sepsis, and sampling department. A multivariate analysis was performed to elucidate the critical factors, which provided directions for future research. To the best of our knowledge, this study is the first Taiwanese study on multiple factors of blood culture positivity rate.

In conclusion, our research revealed that the total blood culture volume and the diagnosis of sepsis were critical factors for the positivity rate of blood culture in the era of automated continuous monitoring of blood culture systems. In addition, insufficient blood volume in each bottle was the main factor affecting the positivity rate. Improving this aspect of sample collection could be the key to increasing positivity rate.

## Acknowledgments

The authors would like to acknowledge staff in microbiology laboratory of Department of Clinical Pathology at Far Eastern Memorial Hospital for facilitating the study. This study was supported by research grants from Far Eastern Memorial Hospital, New Taipei City, Taiwan (FEMH-2021-C-082).

## Author contributions

Conceptualization: Pei-Chin Lin, Chia-Ling Chang, Fang-Yeh Chu; Data curation: Pei-Chin Lin, Chia-Ling Chang, Yi-Hua Chung; Formal analysis: Pei-Chin Lin, Chia-Ling Chang, Yi-Hua Chung, Chih-Chun Chang; Investigation: Pei-Chin Lin, Chia-Ling Chang, Yi-Hua Chung; Methodology: Pei-Chin Lin, Chia-Ling Chang, Chih-Chun Chang, Fang-Yeh Chu; Software: Pei-Chin Lin, Chia-Ling Chang, Chih-Chun Chang; Supervision: Fang-Yeh Chu; Validation: Fang-Yeh Chu; Writing—original draft: Pei-Chin Lin, Chia-Ling Chang; Writing—review & editing: Pei-Chin Lin, Chia-Ling Chang, Yi-Hua Chung, Chih-Chun Chang, Fang-Yeh Chu.

## Footnotes

* Other Gram-positive bacteria included *Enterococcus casseliflavus, Clostridium perfringens, Streptococcus pneumoniae, Clostridium septicum, Corynebacterium species, Corynebacterium striatum, Dermabacter hominis, Micrococcus luteus, β-Streptococcus group A, Corynebacterium afermentans, Corynebacterium coyleae, Corynebacterium glucuronolyticum, Corynebacterium imitans, Corynebacterium tuberculostearicum, Corynebacterium urealyticum, Enterococcus gallinarum, Finegoldia magna, Gordonia species, Granulicatella elegans, Helcococcus sueciensis, Lactobacillus gasseri, Lactobacillus salivarius, Streptococcus sanguinis*, and *Streptococcus suis*.

† Other Gram-negative bacteria included *Aeromonas hydrophila, Serratia marcescens, Morganella morganii, Bacteroides fragilis, Enterobacter aerogenes, Providencia rettgeri, Brevibacterium ravenspurgense, Propionibacterium acnes, Aggregatibacter segnis, Bacteroides thetaiotaomicron, Bacteroides vulgatus, Bilophila wadsworthia, Klebsiella variicola, Pantoea septica, Parabacteroides goldsteinii, Peptoniphilus harei, Prevotella disiens, Proteus vulgaris, Pseudomonas stutzeri, Robinsoniella peoriensis, Sphingobacterium spiritivorum*, and *Sphingomonas paucimobilis*.

‡ Other yeasts included *Candida guilliermondii* and *Candida krusei*.
